# Prudent Use of Blood Cultures for Hospitalized Patients With Cirrhosis

**DOI:** 10.7759/cureus.65389

**Published:** 2024-07-25

**Authors:** Muhammad Shafiq, Muhammad K Amin, Muhammad A Khan

**Affiliations:** 1 Internal Medicine, University of Kansas Medical Center, Kansas City, USA

**Keywords:** database, outcomes, bacteremia, hospitalization, blood cultures, cirrhosis

## Abstract

Background

No reliable risk stratification method is available to guide the extent of infectious work-up among hospitalized patients with cirrhosis. Therefore, we aimed to create a risk stratification method for obtaining blood cultures from hospitalized patients with cirrhosis.

Methods

This was a retrospective cohort study using the Healthcare Cost and Utilization Project - National Readmission Database 2019. Adult patients who were not immunocompromised comprised the final cohort. The primary outcome was the incidence of bacteremia among hospitalized patients with cirrhosis. Secondary outcomes included length of hospital stay, inpatient mortality, and 30-day readmission rate among cirrhosis patients with and without bacteremia. After propensity score matching, the *χ^2^* test was used to assess the primary outcome and inpatient mortality. The Wilcoxon signed-rank test was used to compare the length of hospital stay. Readmission rates were compared *via* survival analysis. Concomitant bacterial infection, cirrhosis causes, and complications were assessed as potential risk factors for bacteremia using binomial regression.

Results

The risk ratio (RR) of bacteremia was 1.66 (95% confidence interval (CI): 1.55-1.78) among patients with cirrhosis compared to those without cirrhosis. A concomitant bacterial infection was found to have a strong association with bacteremia in patients with cirrhosis (RR: 3.3, 95% CI: 3.03-3.59). Among cirrhosis patients without concomitant bacterial infection, the incidence of bacteremia was 0.76% (<1%). Among the causes of cirrhosis, primary sclerosing cholangitis was found to have a strong association with bacteremia (RR: 3.88, 95% CI: 2.3-6.04, P < 0.001). Patients with cirrhosis who had bacteremia were hospitalized three days longer than those without bacteremia. There was no difference in inpatient mortality or 30-day readmission rates between cirrhotic patients with and without bacteremia.

Conclusion

This study suggests that, in the absence of another concomitant bacterial infection and primary sclerosing cholangitis, we can avoid unnecessary blood cultures among immunocompetent patients with cirrhosis. However, given some inherent limitations associated with the database (such as the unavailability of vitals or laboratory values), additional studies are needed to validate its findings.

## Introduction

Liver cirrhosis is the most common chronic liver disease, with a significant mortality burden [1]. In 2021, chronic liver disease and cirrhosis were ranked ninth by the Centers for Disease Control in terms of mortality in the United States. Among hospitalized patients with cirrhosis, the incidence of infection has been reported to be approximately 25-35%, with bacteria being the most common pathogen [2]. Bacterial infections in patients with cirrhosis are associated with higher morbidity and mortality when compared to patients without cirrhosis [3]. Therefore, infectious work-up and timely management of infections are of paramount importance among hospitalized patients with cirrhosis.

Common bacterial infections among patients with cirrhosis include urinary tract infection (UTI), spontaneous bacterial peritonitis (SBP), community-acquired pneumonia, and bacteremia [4]. Bacteremia has been associated with numerous concomitant infections, including SBP, UTI, community-acquired pneumonia, and others [2,3]. Among bacterial infections, bacteremia has been known to be associated with the worst morbidity, including increased length of stay, excessive cost, and increased mortality [5].

Currently, there is no reliable risk stratification method available to guide the extent of infectious work-up among patients with cirrhosis who are hospitalized. There is also no reliable guidance regarding the proper utilization of blood cultures in patients with cirrhosis. As much as infectious work-ups are important, ordering blood cultures for every patient with cirrhosis can be counterproductive as it can lead to unnecessary discomfort for the patient, increase the cost, and prolong the hospital stay by having to wait at least 48 hours for blood cultures to remain negative. To ensure patient safety and proper utilization of blood cultures, we aimed to create a risk stratification method for obtaining blood cultures from patients with cirrhosis who are hospitalized using the Healthcare Cost and Utilization Project - National Readmission Database (HCUP-NRD) 2019.

## Materials and methods

Study design

This was a retrospective cohort study using the HCUP-NRD 2019. The International Classification of Diseases, 10th Revision diagnostic codes were used to define the computable phenotypes for cirrhosis, immunosuppression status, baseline comorbidities (such as hypertension), causes of cirrhosis, complications related to cirrhosis (such as ascites), presence of bacterial infection other than bacteremia (such as pneumonia), bacteremia, and the causative agent of bacteremia. Once the computable phenotypes were defined for the aforementioned elements, the "dplyr" package in R software version 4.3.3 (The R Foundation, Indianapolis, IN, USA) was used to extract data from the HCUP-NRD 2019. Details regarding the exact definitions of the computable phenotypes and methods of their extraction from the HCUP-NRD 2019 are provided in the Appendix.

Data regarding readmissions, age, sex, month of admission, length of hospital stay, and inpatient mortality were directly provided by the HCUP-NRD 2019.

Selection criteria

The inclusion criteria included all patients aged 18 years and older. Immunosuppression independently increases the risk of infection, including bacteremia; therefore, those patients who had a condition that warranted immunosuppression (for instance, renal transplant status) or in whom the condition itself suggested immunosuppression (for instance, neutropenia) or who were using medications that can cause immunosuppression (such as biologic therapy) were excluded from this study. A detailed list of conditions or therapies that suggest or cause immunosuppression and were used to exclude patients is given in the Appendix.

Since autoimmune hepatitis, primary biliary cholangitis, and primary sclerosing cholangitis can cause cirrhosis, patients with these conditions were not excluded. Binomial regression was used to assess the association between bacteremia and the cause of cirrhosis, including autoimmune hepatitis, primary biliary cholangitis, and primary sclerosing cholangitis, as further discussed below.

Groups

Once the final cohort was selected, patients were divided into two groups: patients with cirrhosis and patients without cirrhosis.

Outcomes

The primary outcome was the incidence of bacteremia among hospitalized patients with cirrhosis when compared to patients without cirrhosis. The secondary outcomes included: (1) length of hospital stay of cirrhosis patients with bacteremia and without bacteremia; (2) incidence of inpatient mortality among cirrhosis patients with bacteremia and without bacteremia; and (3) 30-day readmission rates among cirrhosis patients with bacteremia and without bacteremia who were admitted during the first 11 months and were discharged alive. If a patient was admitted during the 12th month, the 30-day readmission outcome could not be calculated for such a patient in the HCUP-NRD 2019.

Hospitalizations

If a patient had more than one hospitalization recorded in the HCUP-NRD 2019, only the first hospitalization was assessed for all outcomes, except the 30-day readmission outcome. For the readmission outcome, only the time to first readmission was assessed (if there was a readmission encounter/hospitalization).

Statistical analysis

In the study population, patients who had a diagnosis of cirrhosis were identified. Patients with cirrhosis were then matched with patients without cirrhosis on age, sex, and baseline comorbidities, including congestive heart failure, ischemic heart disease, hypertension, hyperlipidemia, diabetes mellitus, chronic kidney disease/end-stage renal disease, chronic obstructive pulmonary disease/emphysema, and asthma. Propensity score matching (PSM) with the nearest neighbor method at a 1:1 ratio and without replacement was used for matching. The caliper was set at 0.1 for PSM.

After PSM was complete, the χ2 test was used to compare the incidence of bacteremia between patients with and without a diagnosis of cirrhosis (primary outcome). Both the incidence rate and risk ratio (RR) were calculated for bacteremia. Among patients with cirrhosis, infections other than bacteremia, upper GI bleeding, ascites, portal hypertension, encephalopathy, hepatocellular carcinoma, hepatorenal syndrome, and hepatopulmonary syndrome were subsequently evaluated as potential risk factors for bacteremia using binomial regression. The association between the cause of cirrhosis and bacteremia was also assessed via binomial regression among patients with cirrhosis. All binomial regression assumptions were assessed, including assumptions of no multicollinearity. To ensure model adequacy, the Hosmer-Lemeshow goodness-of-fit test was also performed.

As the duration of hospital stay did not have a normal distribution, the Wilcoxon signed-rank test was used to compare the duration of hospital stay between cirrhosis patients with and without bacteremia. Inpatient mortality between cirrhosis patients with and without bacteremia was compared via the χ2 test, and the mortality rate and RR were calculated.

Once patients were selected for the 30-day readmission outcome, PSM was used first to match patients with and without bacteremia based on age, sex, baseline comorbidities, and complications of cirrhosis (including upper GI bleeding, ascites, portal hypertension, encephalopathy, hepatocellular carcinoma, hepatorenal syndrome, and hepatopulmonary syndrome). After PSM was complete, survival analysis was performed to compare the readmission rates between cirrhotic patients with and without bacteremia. For survival analysis, a Kaplan‒Meier survival curve was constructed, and a Cox proportional hazards model was used to calculate the hazard ratio. The proportional hazards assumption was assessed using the Schoenfeld proportionality test.

The alpha criterion was set at 0.05 for all statistical tests. Weighted analysis was not performed because this study focused on comparative analysis between two groups rather than calculating the national estimates. R software was used for data extraction, data cleaning, and analysis.

## Results

**Figure 1:**
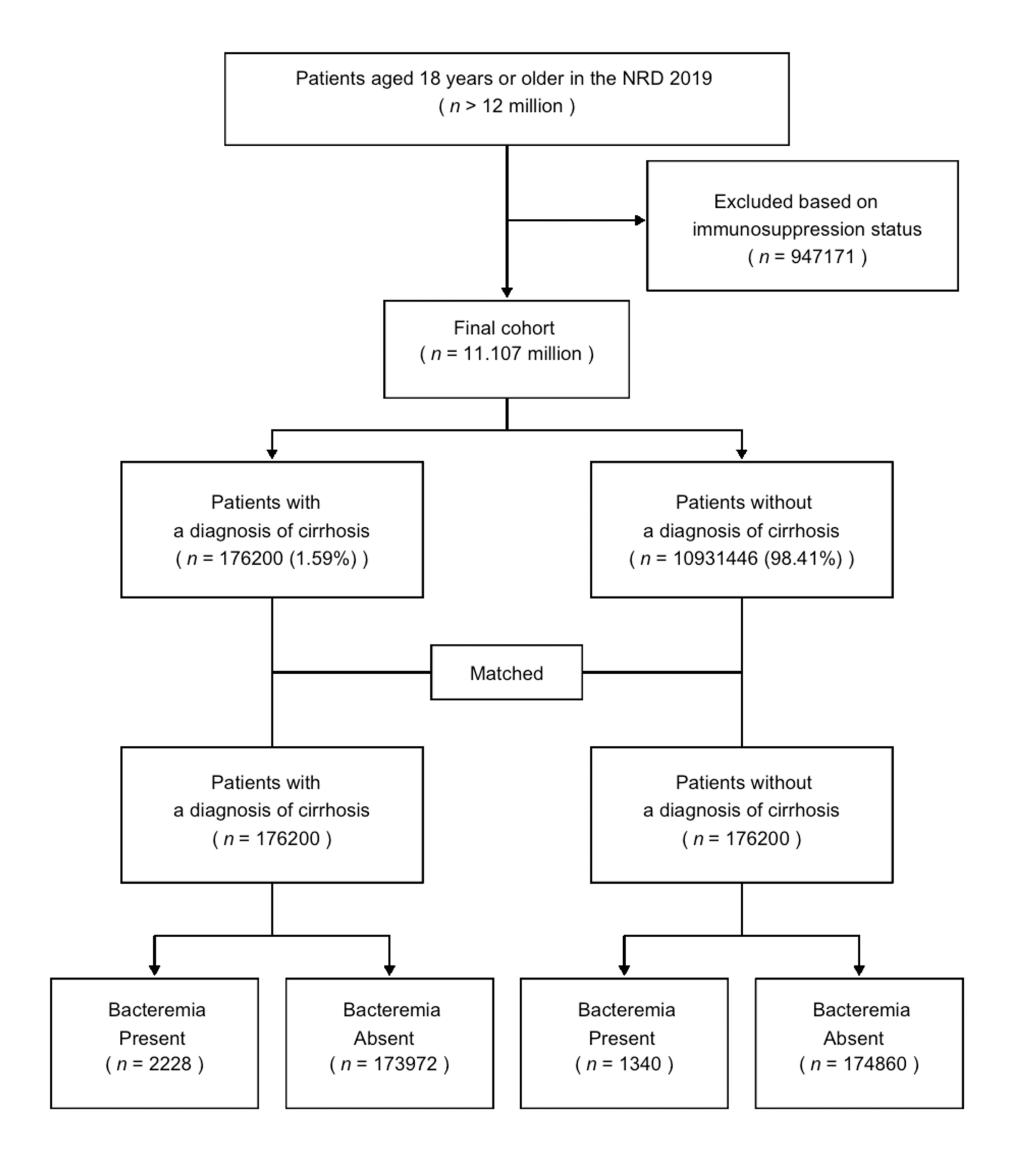
Flowchart of the cohort selection NRD: National Readmission Database

There are more than 12 million unique adult patients in the HCUP-NRD 2019. Of those patients, 11.107 million patients were included in the final cohort, as illustrated in Figure 1.

Once the final cohort was selected, patients with cirrhosis were then matched with patients without cirrhosis using PSM, as summarized in Table 1.

**Table 1:** Demographics and baseline characteristics of patients with and without cirrhosis before and after PSM CHF: congestive heart failure; CKD: chronic kidney disease; COPD: chronic obstructive pulmonary disease; DM: diabetes mellitus; ESRD: end-stage renal disease; HLD: hyperlipidemia; HTN: hypertension; IHD: ischemic heart disease; SD: standard deviation; PSM: propensity score matching

Demographics and baseline comorbidities	Before matching			After matching		
	Cirrhosis present, n = 176200	Cirrhosis absent, n = 10931446	P-value	Cirrhosis present, n = 176200	Cirrhosis absent, n = 176200	P-value
Age in yr, mean (SD)	61.87 (12.86)	56.64 (21.00)	<0.001	61.87 (12.86)	61.93 (12.89)	0.196
Sex = male (%)	107350 (60.9)	4419028 (40.4)	<0.001	107350 (60.9)	107426 (61.0)	0.796
CHF = yes (%)	43472 (24.7)	1713140 (15.7)	<0.001	43472 (24.7)	43280 (24.6)	0.455
IHD = yes (%)	34183 (19.4)	2124539 (19.4)	0.715	34183 (19.4)	34153 (19.4)	0.902
HTN = yes (%)	114590 (65.0)	5847451 (53.5)	<0.001	114590 (65.0)	115029 (65.3)	0.121
HLD= yes (%)	52387 (29.7)	3886927 (35.6)	<0.001	52387 (29.7)	52575 (29.8)	0.491
DM = yes (%)	64033 (36.3)	2655868 (24.3)	<0.001	64033 (36.3)	64122 (36.4)	0.758
CKD/ESRD = yes (%)	44008 (25.0)	1674309 (15.3)	<0.001	44008 (25.0)	43680 (24.8)	0.203
COPD/emphysema = yes (%)	34395 (19.5)	1478853 (13.5)	<0.001	34395 (19.5)	34318 (19.5)	0.747
Asthma = yes (%)	10124 (5.7)	830555 (7.6)	<0.001	10124 (5.7)	9960 (5.7)	0.236

Primary outcome

After PSM was complete for patients with and without cirrhosis, the incidence of bacteremia was compared between the two groups, which was the primary outcome of this study. The summary of the primary outcome is presented in Table 2.

**Table 2:** Primary outcome CI: confidence interval, RR: risk ratio

Cirrhosis	Bacteremia		Total	Incidence rate	RR (95%CI)	P-value
	Present	Absent				
Present	2228	173972	176200	1.26%	1.66 (1.55, 1.78)	<0.01
Absent	1340	174860	176200	0.76%		

Exploratory analysis results

Out of 176200 patients with cirrhosis who were included for analysis after the PSM, 59% (n = 103840) had at least one important complication secondary to cirrhosis, including ascites, SBP, upper GI bleeding, encephalopathy, hepatorenal syndrome, hepatopulmonary syndrome, hepatocellular carcinoma, or portal hypertension. Besides the presence of infection other than bacteremia, these aforementioned complications were included in the binomial regression to assess their association with bacteremia. In binomial regression, the presence of infection other than bacteremia had a strong association with bacteremia among patients with cirrhosis, with an RR of 3.3 (95% confidence interval (CI): 3.03-3.59, P < 0.001). A summary of this binomial regression is presented in Table 3. The binomial regression model met all assumptions, including the assumption of no multicollinearity, and the model appeared to fit the data adequately (Hosmer‒Lemeshow goodness-of-fit test, P > 0.05).

**Table 3:** Summary of binomial regression for potential risk factors for bacteremia among patients with cirrhosis * present CI: confidence interval; RR: risk ratio; GI: gastrointestinal

Potential risk factor*	RR	95% CI	P-value
Presence of infection other than bacteremia	3.30	3.03-3.59	<0.001
Upper GI bleeding	0.78	0.68-0.89	<0.001
Ascites	1.10	1.01-1.20	0.033
Portal hypertension	1.27	1.16-1.40	<0.001
Encephalopathy	1.20	1.06-1.36	0.003
Hepatocellular carcinoma	0.94	0.76-1.15	0.580
Hepatorenal syndrome	1.00	0.82-1.21	0.990
Hepatopulmonary syndrome	1.25	0.58-2.32	0.520

Based on the results of the binomial regression, further exploratory analysis was performed to assess the incidence of bacteremia among patients with cirrhosis who had no other infection. The incidence of bacteremia among patients with cirrhosis in the absence of another identifiable infection (such as UTI or SBP) was only 0.76% (n = 972).

Alcohol use was found to be the most common cause of cirrhosis (36.59%, n = 64465), followed by chronic hepatitis C infection (9.93%, n = 17492) and the simultaneous presence of alcohol use and chronic hepatitis C infection (7.6%, n = 13387). The exact etiology of cirrhosis could not be identified in 26.97% (n = 47522) of patients with cirrhosis. A summary of the causes of cirrhosis is provided in Table 4. Among the causes of cirrhosis, primary sclerosing cholangitis was found to have a strong association with bacteremia, with an RR of 3.88 (95% CI: 2.3-6.04, P < 0.001). Chronic hepatitis C infection was also found to have a statistically significant association with bacteremia, but the strength of association was weak, which was likely related to the large power because of the large sample size (RR of 1.19 with a 95% CI of 1.08-1.32).

**Table 4:** Causes of cirrhosis

Cause of cirrhosis	Count	Percentage
Alcohol use	64465	36.59
Unspecified	47522	26.97
Chronic hepatitis C	17492	9.93
Alcohol use + chronic hepatitis C	13387	7.60
Nonalcoholic steatohepatitis	13035	7.40
Cardiac	8482	4.81
Primary biliary cholangitis	2303	1.31
Two causes (other than specified)	1907	1.08
Chronic hepatitis B	1743	0.99
Alcohol use + nonalcoholic steatohepatitis	1480	0.84
Autoimmune hepatitis	1188	0.67
More than two causes	1015	0.58
Alcohol use + chronic hepatitis B	680	0.39
Chronic hepatitis B + chronic hepatitis C	680	0.39
Chronic hepatitis C + nonalcoholic steatohepatitis	389	0.22
Primary sclerosing cholangitis	152	0.09
Chronic hepatitis B + nonalcoholic steatohepatitis	103	0.06
Cystic fibrosis	70	0.04
Hereditary hemochromatosis	70	0.04
Wilson disease	37	0.02

In 43.63% (n = 972) of cirrhosis patients with bacteremia, another concomitant infection could not be identified in this study. Among the concomitant infections that could be identified along with bacteremia, UTI was the most common infection (12.16%, n = 271), followed by cellulitis/soft tissue infections (9.07%, n = 202) and bacterial pneumonia (8.75%, n = 195).

In 42.41% (n = 945) of cirrhosis patients with bacteremia, the exact bacterial agent responsible for bacteremia could not be identified. Among the remaining 57.59% (n = 1283) cirrhosis patients with bacteremia, the exact bacterial agent could be identified. Among those 1283 cirrhosis patients with bacteremia, in whom the exact bacterial agent could be identified, *Staphylococcus aureus* (*S. aureus*) was responsible for bacteremia in 28.60% (n = 367) of patients, followed by all groups of *Streptococcus* (24.24%, n = 311) and *Escherichia coli* (*E. coli*) (23.23%, n = 298).

Secondary outcomes

Cirrhosis patients with bacteremia were hospitalized for a longer duration than patients without bacteremia, with a median hospital length of stay of seven days compared to four days, respectively (median difference: three days, 95% CI: 2.99-3.00, P < 0.001). The inpatient mortality rates for cirrhosis patients with and without bacteremia were 6.78% (n = 151) and 7.17% (n = 12469), respectively. There was no significant difference in mortality between these two groups, with an RR of 0.95 (95% CI: 0.81-1.10, P = 0.48).

Before determining readmission outcomes for patients with cirrhosis who were discharged alive, patients with cirrhosis who had bacteremia and those who did not were matched as well. A summary of this PSM is provided in Table 5.

**Table 5:** Demographics, baseline characteristics, and complications of patients with cirrhosis with and without bacteremia before and after PSM CAD: coronary artery disease; CHF: congestive heart failure; CKD: chronic kidney disease; COPD: chronic obstructive pulmonary disease; DM: diabetes mellitus; ESRD: end-stage renal disease; GI: gastrointestinal; HCC: hepatocellular carcinoma; HLD: hyperlipidemia; HPS: hepatopulmonary syndrome; HRS: hepatorenal syndrome; HTN: hypertension; SBP: spontaneous bacterial peritonitis; SD: standard deviation; PSM: propensity score matching

Characteristics	Before matching			After matching		
	Bacteremia present, n = 1934	Bacteremia absent, n = 149821	P-value	Bacteremia present, n = 1933	Bacteremia absent, n = 1933	P-value
Age in yr, mean (SD)	61.90 (12.75)	61.68 (12.84)	0.452	61.90 (12.75)	61.87 (12.66)	0.946
Sex = male (%)	1273 (65.8)	90820 (60.6)	< 0.001	1272 (65.8)	1295 (67.0)	0.454
CHF = yes (%)	483 (25.0)	36449 (24.3)	0.528	482 (24.9)	450 (23.3)	0.244
CAD = yes (%)	361 (18.7)	29337 (19.6)	0.327	361 (18.7)	357 (18.5)	0.901
HTN = yes (%)	1248 (64.5)	98989 (66.1)	0.162	1247 (64.5)	1259 (65.1)	0.711
HLD = yes (%)	542 (28.0)	45644 (30.5)	0.022	542 (28.0)	526 (27.2)	0.590
DM = yes (%)	733 (37.9)	55469 (37.0)	0.441	733 (37.9)	750 (38.8)	0.597
CKD/ESRD = yes (%)	556 (28.7)	37251 (24.9)	< 0.001	555 (28.7)	549 (28.4)	0.859
COPD/emphysema = yes (%)	344 (17.8)	29418 (19.6)	0.045	344 (17.8)	337 (17.4)	0.800
Asthma = yes (%)	125 (6.5)	9037 (6.0)	0.457	125 (6.5)	102 (5.3)	0.132
Ascites = yes (%)	787 (40.7)	52586 (35.1)	< 0.001	786 (40.7)	747 (38.6)	0.212
SBP = yes (%)	133 (6.9)	3643 (2.4)	< 0.001	132 (6.8)	131 (6.8)	1.000
Upper GI bleeding = yes (%)	187 (9.7)	20690 (13.8)	< 0.001	187 (9.7)	166 (8.6)	0.264
Encephalopathy = yes (%)	239 (12.4)	11312 (7.6)	< 0.001	238 (12.3)	225 (11.6)	0.552
HRS = yes (%)	83 (4.3)	4060 (2.7)	< 0.001	83 (4.3)	70 (3.6)	0.322
HPS = yes (%)	7 (0.4)	329 (0.2)	0.280	7 (0.4)	7 (0.4)	1.000
HCC = yes (%)	79 (4.1)	6602 (4.4)	0.529	79 (4.1)	64 (3.3)	0.233
Portal HTN = yes (%)	637 (32.9)	43134 (28.8)	< 0.001	636 (32.9)	615 (31.8)	0.492

**Figure 2:**
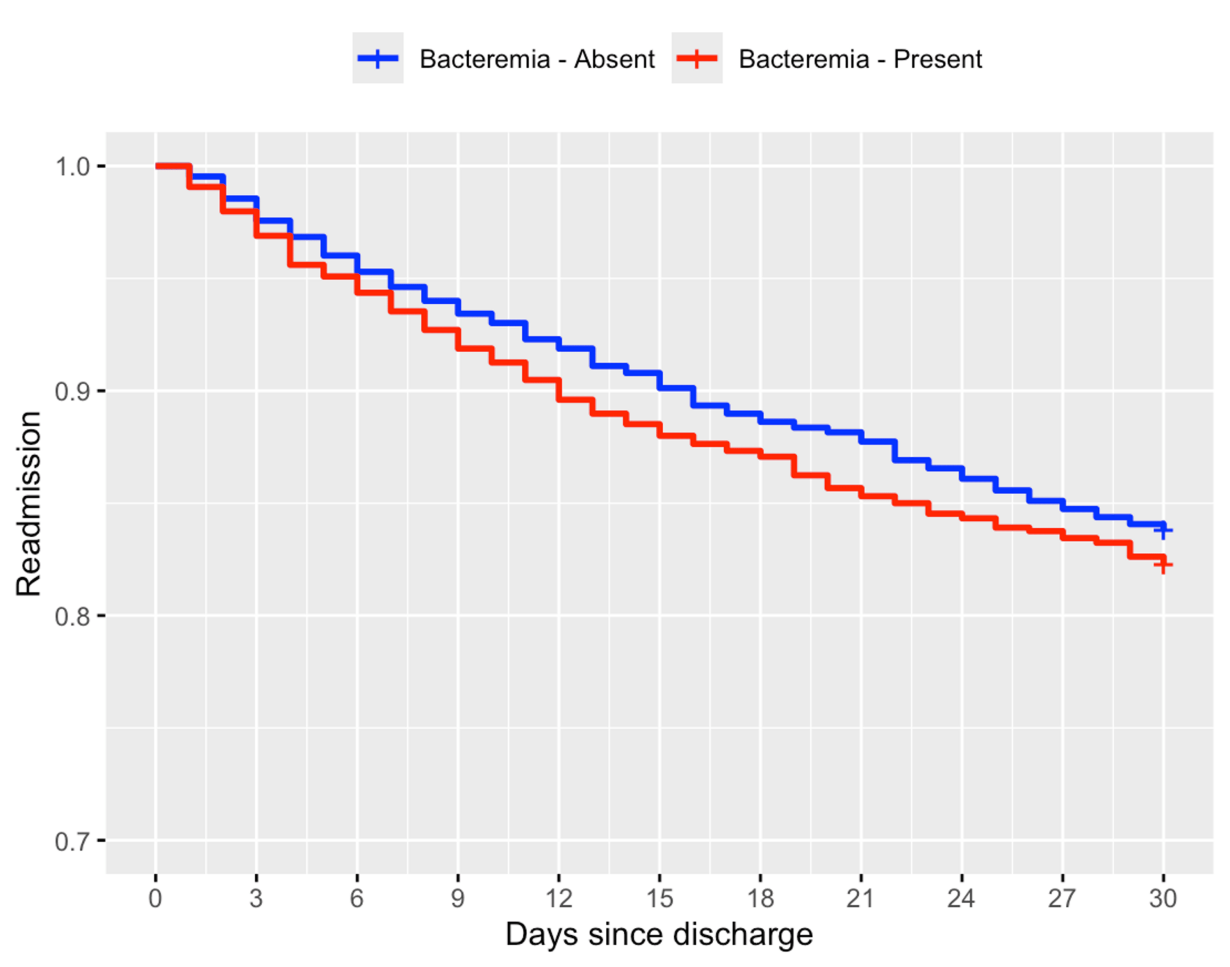
Kaplan-Meier survival curve for 30-day readmission outcome between cirrhosis patients with and without bacteremia

After PSM, survival analyses were performed. As shown in Figure 2, the Kaplan-Meier survival plot does not show any significant deviation in readmission rates between cirrhosis patients with and without bacteremia.

The hazard ratio for the 30-day readmission rate between cirrhosis patients with and without bacteremia was also 1.11 (95% CI: 0.95-1.29, P = 0.200). Hence, there was no significant difference between cirrhosis patients with and without bacteremia in regard to the 30-day readmission rate. The proportional hazards assumption was met (Schoenfeld proportionality test, P > 0.05).

## Discussion

This study found the risk of bacteremia to be 1.66 times higher among patients with cirrhosis than among those without cirrhosis. The presence of an infection other than bacteremia (such as UTI, SBP, or pneumonia) was found to have a strong association with bacteremia among patients with cirrhosis, with an RR of 3.3 (95% CI: 3.03-3.59, P < 0.001). This association suggests that the higher incidence of bacteremia may be driven by the higher incidence of concomitant bacterial infections among patients with cirrhosis. Among cirrhosis patients without another concomitant infection, the incidence of bacteremia was similar to that among patients without cirrhosis (0.76% vs. 0.76%, respectively).

The most common cause of cirrhosis in this study was alcohol use. Among the causes of cirrhosis, primary sclerosing cholangitis was only found in 0.09% (n = 152) of patients, but it was found to have a strong association with bacteremia, with an RR of 3.88 (95% CI: 2.30-6.04, P < 0.001). Among cirrhosis patients with bacteremia, the most common concomitant infections were UTIs, followed by cellulitis and bacterial pneumonia. The most common bacterial agent responsible for bacteremia that could be identified in our study was *S. aureus*, followed by *Streptococcus* (all groups) and *E. coli*. Patients with cirrhosis who had bacteremia were hospitalized three days longer than those without bacteremia. There was no difference in inpatient mortality or 30-day readmission rates between cirrhosis patients with and without bacteremia in our study.

Patients with cirrhosis are vulnerable to bacterial infections [6-8]. This is evident by the findings of our study as well, in which bacteremia was noted to have a higher incidence among patients with cirrhosis than among patients without cirrhosis. According to the published literature, bacterial infections account for at least 25% of hospital admissions among patients with cirrhosis and are considered an important factor in decompensation [9,10]. Potential causes that lead to a higher incidence of bacterial infections among patients with cirrhosis include altered intestinal permeability, changes in gut microflora, and alterations in innate immunity and humoral immunity [9,10]. Other described mechanisms include decreased hepatic complement production, impaired Kupffer cells, altered neutrophil chemotaxis, and downregulated HLA-DR expression on monocytes [11,12].

Bacterial infections in cirrhosis are associated with a high incidence of sepsis and related mortality [13]. Other factors linked with hospital mortality in cirrhosis include hepatorenal syndrome, hepatic encephalopathy, advanced age, renal insufficiency, and admission to the intensive care unit [14]. High white blood cell count, multi-organ failure, and initiation of dialysis for renal failure in liver failure also have a grave prognosis. Our study also found that patients with cirrhosis who had UTIs, cellulitis, pneumonia, SBP, or any other bacterial infection had a significantly increased risk of bacteremia. However, this study did not find any difference in inpatient mortality among cirrhosis patients with and without bacteremia. This may be the result of the broader use of empiric antibiotics for patients with cirrhosis in the recent past. Similarly, we (the authors) also believe that the broader use of empiric antibiotics among cirrhosis patients with GI bleeding may be the reason why those patients with GI bleeding had a low incidence of bacteremia in our study. However, we could not ascertain this fact because the HCUP-NRD does not provide data on medication administration.

Gram-negative bacteria, namely *E. coli*, *Klebsiella*, and *Aeromonas* have been the most reported organisms in culture-positive hospitalized patients with cirrhosis (>70%) [6,15]. Gram-positive bacteria, predominantly *Streptococcus* and *Staphylococcus*, were reported in 21.2% of patients in a relevant study by Kuo et al. [15]. Among the identified pathogens in our study, *S. aureus *and *Streptococcus* (all groups) represented the most common pathogens responsible for bacteremia in patients with cirrhosis. Collectively, our study findings do correlate with those of previous studies in which *E. coli*, *Staphylococcus*, and *Streptococcus *represent the bulk of causative agents responsible for bacteremia among patients with cirrhosis.

In the literature, common sources linked to bacteremia are SBP, UTI, pneumonia, and biliary tree infection [4,16]. Multiple other studies have mentioned UTI as a predictor of other bacterial infections in patients with cirrhosis [17-19]. In a study by Choudry et al. [20], Gram-positive bacteria were prevalent in patients with SBP, whereas Gram-negative organisms were isolated in UTIs and pneumonia. Although SBP has been widely reported to be one of the leading causes of bacteremia among patients with cirrhosis, this was not the case in our study. In our study, the most common infections associated with bacteremia in patients with cirrhosis were UTIs, followed by cellulitis and bacterial pneumonia. This is in part in agreement with the findings of previous studies, as this study agreed on UTI and pneumonia as important risk factors for bacteremia.

Because of the higher incidence of bacterial infections and associated worse outcomes, a broad infectious work-up is usually ordered for all patients with cirrhosis on admission, including blood cultures, even if the patient does not have evidence of any concomitant infection. Although it is of the utmost importance to perform appropriate infectious work-up, currently no reliable stratification method exists to guide the proper utilization of blood cultures among hospitalized patients with cirrhosis. Our study undertook this challenge to provide at least some guidance on the appropriate use of blood cultures for patients with cirrhosis. If there is no concomitant infection, the incidence of bacteremia is less than 1%, according to the findings of our study. Therefore, among patients with cirrhosis who do not have any objective evidence of concomitant bacterial infection (such as SBP, UTI, pneumonia, or cellulitis), who are not immunocompromised, in whom the cause of cirrhosis is not primary sclerosing cholangitis, and who do not have any other signs/symptoms to suggest an infection (such as fever), we can avoid unnecessary blood cultures.

There were limitations to this study. This study was based on the HCUP-NRD, which does not provide data on vitals, laboratory values, or medication administration. This limited our ability to determine the severity of cirrhosis (as we could not calculate the model for End-Stage Liver Disease or the Child-Pugh score). To counter this limitation, the International Classification of Diseases, 10th Revision codes were used to identify important complications of cirrhosis, including ascites, portal hypertension, encephalopathy, hepatocellular carcinoma, hepatorenal syndrome, and hepatopulmonary syndrome. Since these complications suggest decompensated cirrhosis, binomial regression was used to assess their association with bacteremia, as summarized in Table 3. We expected some variability in billing codes for the same diagnosis between different providers; for example, hypertension may be billed as I10 or I16. To counter this shortcoming, we used meticulously defined computable phenotypes, as detailed in the Appendix. The HCUP-NRD also does not provide data on out-of-hospital mortality.

## Conclusions

Among patients with cirrhosis who do not have any objective evidence of concomitant bacterial infection, who are not immunocompromised, in whom the cause of cirrhosis is not primary sclerosing cholangitis, and who do not have any other signs/symptoms to suggest an infection (such as fever), we can avoid unnecessary blood cultures. This can avoid unnecessary discomfort for the patients, reduce unnecessary costs, and reduce the length of the hospital stay. However, considering the inherent limitations of this study, providers should exercise caution, especially if a cirrhosis patient has severe disease (e.g., Child Class C or Model for End-Stage Liver Disease - Sodium Score >24). Further prospective studies are crucial for the validation of the conclusion of this study, and in those studies, it will be important to include data on the severity of liver disease as well as prior antibiotic use.

## Appendices

International Classification of Diseases, 10th Revision (ICD-10) diagnostic codes were used to define the computable phenotypes for cirrhosis, immunosuppression status, baseline comorbidities, causes of cirrhosis, complications related to cirrhosis, presence of bacterial infections other than bacteremia, bacteremia, and the causative agent of bacteremia. A description of each element is given below.

We divided the ICD-10 codes into two types: specific and broad. A specific code means that it is a billable code and doesn’t require any further details. Broad code means that it is a parent/general code and is nonspecific. Many specific codes fall under the broad code. For example, K70.3 is a more general/broad code for alcoholic cirrhosis of the liver. This is a non-billable code because it is not specific, as it doesn’t provide any details regarding the presence or absence of ascites. Two more specific codes fall under this broad code, which are K70.30 and K70.31. K70.30 represents alcoholic cirrhosis of the liver without ascites, while K70.31 represents alcoholic cirrhosis of the liver with ascites.

If we needed all of the specific codes that fall under a broad code, for efficiency, we used the broad ICD-10 code, and that is why we divided the ICD-10 codes into these two categories for our study. For example, we needed ICD-10 codes to identify patients with chronic kidney disease (CKD) or end-stage renal disease (ESRD) status. Instead of using specific ICD-10 codes for each stage of CKD (such as N18.1 for CKD stage -1 or N18.2 for CKD stage -2), we used the broad code of N18 in our search, and it captured all forms of CKD as well as ESRD status.

We used the "dplyr" package in R software version 4.3.3 (The R Foundation, Indianapolis, IN, USA) to extract data from the National Readmission Database (NRD) 2019 in this study, using definitions for each element as further detailed below.

Cirrhosis

If the patient had any of the ICD-10 codes listed in Table 6 during each hospitalization, then it was considered that the patient had a diagnosis of cirrhosis. For example, Mr. A was hospitalized in January 2019, and there was a billing code for K70.30 during the same hospitalization. Mr. A was admitted again in May 2019, and there was a billing code for K70.31 during his second hospitalization. Mr. A had his third hospitalization in August 2019, and during his third hospitalization, he had a billing code for K70.31 as well. In this scenario, Mr. A was admitted to the hospital three times in 2019, and during each hospitalization, there was at least one billing code for cirrhosis (from Table 6). So, Mr. A was identified as having a diagnosis of cirrhosis.

**Table 6:** ICD-10 codes used for cirrhosis ICD-10: International Classification of Diseases, 10th Revision

S. No	Condition	ICD-10 code	Code type	Description
1	Cirrhosis	K70.30	Specific	Alcoholic cirrhosis of the liver without ascites
		K70.31	Specific	Alcoholic cirrhosis of the liver with ascites
		K71.7	Specific	Toxic liver disease with fibrosis and cirrhosis of the liver
		K74.3	Specific	Primary biliary cholangitis/cirrhosis
		K74.4	Specific	Secondary biliary cirrhosis
		K74.5	Specific	Biliary cirrhosis, unspecified
		K74.6	Broad	Other and unspecified cirrhosis of the liver
		K76.1	Specific	Chronic passive congestion of the liver - applicable to cardiac cirrhosis

In the second scenario, Ms. B was admitted to the hospital in March 2019 and had a billing diagnosis of K74.6. Ms. B was admitted again in May 2019, but during her second hospitalization, there was no billing code for cirrhosis (Table 6). In this second scenario, Ms. B was not considered to have cirrhosis due to inconsistent diagnostic codes between her hospitalizations.

The above definition of cirrhosis is less sensitive but more specific. This was done with the purpose of more accurately identifying the true study population and minimizing the risk of false flagging.

Immunosuppression status

If the patient had any of the ICD-10 codes listed in Table 7 during any hospitalization in 2019, then it was considered that the patient was likely immunosuppressed. For example, Mr. C was admitted to the hospital in March 2019 and July 2019. There was no diagnostic code for liver transplant status (as detailed in Table 7) in the March 2019 hospitalization, but the patient had a billing code of Z94.4 in July 2019. In this example, Mr. C was considered to have a history of liver transplants in 2019 (which requires immunosuppression) and, therefore, was excluded from the study.

**Table 7:** ICD-10 codes for immunosuppression status HIV: human immunodeficiency virus; ICD-10: International Classification of Diseases, 10th Revision

S. No	Condition	ICD-10 code	Code type	Description
1	Liver transplant status	Z94.4	Specific	Liver transplant status
		Z48.23	Specific	Encounter for aftercare following liver transplant
		T86.4	Broad	Complications of liver transplant
2	Kidney transplant status	Z94.0	Specific	Kidney transplant status
		Z48.22	Specific	Encounter for aftercare following kidney transplant
		T86.1	Broad	Complications of kidney transplant
3	Heart transplant status	Z94.1	Specific	Heart transplant status
		Z48.21	Specific	Encounter for aftercare following heart transplant
		T86.2	Broad	Complications of heart transplant
4	Lung transplant status	Z94.2	Specific	Lung transplant status
		Z94.3	Specific	Heart and lung transplant status
		Z48.24	Specific	Encounter for aftercare following lung transplant
		Z48.280	Specific	Encounter for aftercare following heart-lung transplant
		T86.3	Broad	Complications of heart-lung transplant
		T86.81	Broad	Complications of lung transplant
5	Pancreas transplant status	Z94.83	Specific	Pancreas transplant status
6	Systemic steroids use	Z79.52	Specific	Long-term (current) use of systemic steroids
7	Biologics, immunotherapy, and chemotherapy use	Z79.6	Broad	Long-term (current) use of immunomodulators and immunosuppressants
8	Neutropenia	D70	Broad	Neutropenia
9	HIV disease	B20	Specific	HIV disease
10	Crohn's disease	K50	Broad	Crohn's disease
11	Ulcerative colitis	K51	Broad	Ulcerative colitis
12	Rheumatoid arthritis	M05	Broad	Rheumatoid arthritis with rheumatoid factor
		M06	Broad	Other rheumatoid arthritis
13	Sjögren syndrome	M35.0	Broad	Sjögren syndrome
14	Sarcoidosis	D86	Broad	Sarcoidosis
15	Systemic lupus erythematosus	M32	Broad	Systemic lupus erythematosus
16	Systemic sclerosis	M34	Broad	Systemic sclerosis
17	Major/Important vasculitis	M30	Broad	Polyarteritis nodosa and related conditions
		M31.3	Broad	Wegener's granulomatosis
		M31.5	Specific	Giant cell arteritis with polymyalgia rheumatica
		M31.6	Specific	Other giant cell arteritis
		M31.7	Specific	Microscopic polyangiitis
		I77.82	Specific	Antineutrophilic cytoplasmic antibody vasculitis
18	Inflammatory polyneuropathy	G61	Broad	Inflammatory polyneuropathy
19	Dermatopolymyositis	M33	Broad	Dermatopolymyositis
20	Myasthenia gravis	G70.0	Broad	Myasthenia gravis
21	Multiple sclerosis	G35	Specific	Multiple sclerosis
22	History of splenectomy	Z90.81	Specific	Acquired absence of spleen
23	Immune thrombocytopenic purpura	D69.3	Specific	Immune thrombocytopenic purpura

Since autoimmune hepatitis, primary biliary cholangitis, and primary sclerosing cholangitis can cause cirrhosis, these patients were not excluded.

Baseline comorbidities

Baseline comorbidities that have been included in this study are congestive heart failure, ischemic heart disease, hypertension, hyperlipidemia, diabetes mellitus, CKD/ESRD status, chronic obstructive pulmonary disease (COPD)/emphysema, and asthma.

The presence of each baseline comorbidity is defined by the presence of any ICD-10 code from Table 8 during any hospitalization in 2019. For example, Ms. D was hospitalized in January 2019, May 2019, and then again in September 2019. Only during the May 2019 hospitalization did Ms. D have a billing code of I10. Ms. D didn’t have any of the billing codes for hypertension (as listed in Table 8) during her hospitalization in January 2019 and September 2019. In this case, Ms. D was considered to have hypertension because she had a billing code for hypertension at least during one hospitalization in 2019.

**Table 8:** ICD-10 codes for baseline comorbidities CKD: chronic kidney disease; ESRD: end-stage renal disease; STEMI: ST-elevation myocardial infarction; ICD-10: International Classification of Diseases, 10th Revision

S. No	Condition	ICD-10 code	Code type	Description
1	Congestive heart failure	I50	Broad	Heart failure
		I11.0	Specific	Hypertensive heart disease with heart failure
		I13.0	Specific	Hypertensive heart and CKD with heart failure and stage 1 through stage 4 CKD, or unspecified CKD
		I13.2	Specific	Hypertensive heart and CKD with heart failure and with stage 5 CKD, or ESRD
2	Ischemic heart disease	I25.1	Broad	Atherosclerotic heart disease of native coronary artery
		I25.2	Specific	Old myocardial infarction
		I25.5	Specific	Ischemic cardiomyopathy
		I25.6	Specific	Silent myocardial ischemia
		I25.7	Broad	Atherosclerosis of coronary artery bypass graft(s) and coronary artery of transplanted heart with angina pectoris
		I25.8	Broad	Other forms of chronic ischemic heart disease
		I25.9	Specific	Chronic ischemic heart disease, unspecified
		I21.0	Broad	STEMI of the anterior wall
		I21.1	Broad	STEMI of the inferior wall
		I21.2	Broad	STEMI of other sites
		I21.3	Specific	STEMI of unspecified site
		T82.855	Broad	Stenosis of coronary artery stent
		T82.21	Broad	Mechanical complication of coronary artery bypass graft
		Z95.5	Specific	Presence of coronary angioplasty implant and graft
3	Hypertension	I10	Specific	Essential (primary) hypertension
		I11	Broad	Hypertensive heart disease
		I12	Broad	Hypertensive CKD
		I13	Broad	Hypertensive heart and CKD
		I16	Broad	Hypertensive crisis
4	Diabetes mellitus	E10	Broad	Type 1 diabetes mellitus
		E11	Broad	Type 2 diabetes mellitus
5	Hyperlipidemia	E78.0	Broad	Pure hypercholesterolemia
		E78.1	Specific	Pure hyperglyceridemia
		E78.2	Specific	Mixed hyperlipidemia
		E78.49	Specific	Other hyperlipidemia
		E78.5	Specific	Hyperlipidemia, unspecified
6	CKD/ESRD	I12	Broad	Hypertensive CKD
		I13	Broad	Hypertensive heart and CKD
		E10.22	Specific	Type 1 diabetes mellitus with diabetic CKD
		E11.22	Specific	Type 2 diabetes mellitus with diabetic CKD
		N18	Broad	CKD
7	Chronic obstructive pulmonary disease/emphysema	J43	Broad	Emphysema
		J44	Broad	Other chronic obstructive pulmonary disease
8	Asthma	J45	Broad	Asthma

If the patient has many chronic comorbidities and is stable, sometimes clinicians don’t bill for all comorbidities. In contrast to the definition of cirrhosis (as mentioned above), it was the reason why more sensitive but less specific criteria were used to define each baseline comorbidity. For instance, Ms. D has a history of cirrhosis, congestive heart failure, ischemic heart disease, CKD, hyperlipidemia, and hypertension. Ms. D was admitted for acute kidney injury on CKD each time in 2019. Ms. D's blood pressure was within normal range with only one anti-hypertensive medication during all of her three hospitalizations in 2019. A clinician didn’t bill for hypertension in January 2019 and September 2019, but another clinician decided to bill for it in May 2019. This way, Ms. D ended up having a billing code for hypertension only in May 2019. In this scenario, Ms. D was considered to have hypertension for our study.

Causes of cirrhosis

The cause of cirrhosis was identified via the presence of any ICD-10 code from Table 9 during any hospitalization in 2019.

**Table 9:** ICD-10 codes for causes of cirrhosis ICD-10: International Classification of Diseases, 10th Revision

S. No	Condition	ICD-10 code	Code type	Description
1	Chronic hepatitis B	B18.0	Specific	Chronic viral hepatitis B with delta-agent
		B18.1	Specific	Chronic viral hepatitis B without delta-agent
		B19.10	Specific	Viral hepatitis B without hepatic coma
		B19.11	Specific	Viral hepatitis B with hepatic coma
2	Chronic hepatitis C	B18.2	Specific	Chronic viral hepatitis C
		B19.20	Specific	Viral hepatitis C without hepatic coma
		B19.21	Specific	viral hepatitis C with hepatic coma
3	Alcohol use	K70.30	Specific	Alcoholic cirrhosis of the liver without ascites
		K70.31	Specific	Alcoholic cirrhosis of the liver with ascites
4	Cardiac	K76.1	Specific	Chronic passive congestion of the liver - applicable to cardiac cirrhosis
5	Hereditary hemochromatosis	E83.110	Specific	Hereditary hemochromatosis
6	Autoimmune hepatitis	K75.4	Specific	Autoimmune hepatitis
7	Nonalcoholic steatohepatitis	K75.81	Specific	Nonalcoholic steatohepatitis
8	Primary biliary cholangitis	K74.3	Specific	Primary biliary cholangitis, previously called primary biliary cirrhosis
9	Primary sclerosing cholangitis	K83.01	Specific	Primary sclerosing cholangitis
10	Cystic fibrosis	E84.8	Specific	Cystic fibrosis with other manifestations
11	Wilson’s disease	E83.01	Specific	Wilson’s disease

Complications related to cirrhosis and upper GI bleeding including variceal bleeding

Since only the first hospitalization in NRD-2019 was considered to be the primary outcome in this study, only the first hospitalization was assessed for the presence of any billing codes listed in Table 10 to identify each complication and/or upper GI bleeding. For instance, Mr. E had his first hospitalization in April 2019. During this first hospitalization, he had billing codes for I85.01 and R18. Based on these billing codes, the patient was identified as having experienced upper GI bleeding and ascites during his first hospitalization.

**Table 10:** ICD-10 codes for cirrhosis complications and upper GI bleeding GI: gastrointestinal; ICD-10: International Classification of Diseases, 10th Revision; SBP: spontaneous bacterial peritonitis

S. No	Condition	ICD-10 code	Code type	Description
1	SBP	K65.2	Specific	SBP
2	Upper GI bleeding	I85.01	Specific	Esophageal varices with bleeding
		I85.11	Specific	Secondary esophageal varices with bleeding
		K22.11	Specific	Ulcer of the esophagus with bleeding
		K25.0	Specific	Acute gastric ulcer with hemorrhage
		K25.2	Specific	Acute gastric ulcer with both hemorrhage and perforation
		K25.4	Specific	Chronic or unspecified gastric ulcer with hemorrhage
		K25.6	Specific	Chronic or unspecified gastric ulcer with both hemorrhage and perforation
		K26.0	Specific	Acute duodenal ulcer with hemorrhage
		K26.2	Specific	Acute duodenal ulcer with both hemorrhage and perforation
		K26.4	Specific	Chronic or unspecified duodenal ulcer with hemorrhage
		K26.6	Specific	Chronic or unspecified duodenal ulcer with both hemorrhage and perforation
		K29.01	Specific	Acute gastritis with bleeding
		K29.21	Specific	Alcoholic gastritis with bleeding
		K29.31	Specific	Chronic superficial gastritis with bleeding
		K29.41	Specific	Chronic atrophic gastritis with bleeding
		K29.51	Specific	Unspecified chronic gastritis with bleeding
		K29.61	Specific	Other gastritis with bleeding
		K29.71	Specific	Gastritis, unspecified with bleeding
		K29.81	Specific	Duodenitis with bleeding
		K29.91	Specific	Gastroduodenitis, unspecified with bleeding
		K92.0	Specific	Hematemesis
		K92.1	Specific	Melena
3	Encephalopathy	K76.82	Specific	Hepatic encephalopathy
		G93.4	Broad	Other and unspecified encephalopathy
4	Hepatorenal syndrome	K76.7	Specific	Hepatorenal syndrome
5	Hepatopulmonary syndrome	K76.81	Specific	Hepatopulmonary syndrome
6	Ascites	R18	Broad	Ascites
		K70.31	Specific	Alcoholic cirrhosis of the liver with ascites
7	Hepatocellular carcinoma	C22.0	Specific	Liver cell carcinoma
8	Portal hypertension	K76.6	Specific	Portal hypertension

Presence of bacterial infection other than bacteremia

Just like complications related to cirrhosis, only the first hospitalization was assessed for the presence of any billing codes listed in Table 11 to identify the presence of a bacterial infection other than bacteremia.

**Table 11:** ICD-10 codes for the presence of bacterial infection other than bacteremia ICD-10: International Classification of Diseases, 10th Revision; UTI: urinary tract infection

S. No	Condition	ICD-10 code	Code type	Description
1	Pneumonia	J13	Specific	Pneumonia due to *Streptococcus pneumoniae*
		J14	Specific	Pneumonia due to *Hemophilus influenzae*
		J15	Broad	Bacterial pneumonia, not elsewhere classified
		J16.0	Specific	Chlamydia pneumoniae
		J18	Broad	Pneumonia, unspecified organism
2	UTIs (including pyelonephritis)	N10	Specific	Acute pyelonephritis
		N30.0	Broad	Acute cystitis
		N30.8	Broad	Other cystitis
		N30.9	Broad	Cystitis, unspecified
		N34.0	Specific	Urethral abscess
		N34.1	Specific	Nonspecific urethritis
		N34.2	Specific	Other urethritis
		N39.0	Specific	UTI, site not specified
		R82.81	Specific	Pyuria
3	Cellulitis and other skin/soft-tissue infections	L02	Broad	Cutaneous abscess, furuncle, and carbuncle
		L03	Broad	Cellulitis and acute lymphangitis
		K12.2	Specific	Cellulitis and abscess of mouth
		H05.01	Broad	Cellulitis of orbit
		H60.1	Broad	Cellulitis of the external ear
4	Infectious gastroenteritis	A01	Broad	Typhoid and paratyphoid fevers
		A02	Broad	Other salmonella infections
		A03	Broad	Shigellosis
		A04	Broad	Other bacterial intestinal infections
		A09	Specific	Infectious gastroenteritis and colitis, unspecified
5	Bacterial meningitis	G00	Broad	Bacterial meningitis, not elsewhere classified
		G01	Specific	Meningitis in bacterial diseases classified elsewhere
6	Spontaneous bacterial peritonitis	K65.2	Specific	Spontaneous bacterial peritonitis
7	Osteomyelitis (including vertebral osteomyelitis) and discitis	M86	Broad	Osteomyelitis (both acute and chronic)
		M46.2	Broad	Osteomyelitis of vertebra
		M46.3	Broad	Infection of intervertebral disc (pyogenic)
		M46.4	Broad	Discitis, unspecified
8	Endocarditis	I33.0	Specific	Acute and subacute infective endocarditis
9	Septic arthritis	M00	Specific	Pyogenic arthritis
10	Infection of internal orthopedic prosthetic devices	T84.5	Broad	Infection and inflammatory reaction due to internal joint prosthesis
		T84.6	Broad	Infection and inflammatory reaction due to internal fixation device
		T84.7	Broad	Infection and inflammatory reaction due to other internal orthopedic prosthetic devices, implants, and grafts

Bacteremia and causative agent of bacteremia

Just like complications related to cirrhosis, only the first hospitalization was assessed for the presence of any billing codes listed in Table 12 to identify bacteremia, and if bacteremia is present, what is the potential causative agent?

**Table 12:** ICD-10 codes for bacteremia and causative agent of bacteremia ICD-10: International Classification of Diseases, 10th Revision

S. No	Condition	ICD-10 code	Code type	Description
1	Bacteremia	R78.81	Specific	Bacteremia
2	Staphylococcus aureus	B95.6	Broad	*Staphylococcus aureus* is the cause of diseases classified elsewhere
		A41.0	Broad	Sepsis due to *Staphylococcus aureus*
3	Streptococcus	B95.0	Specific	*Streptococcus*, group A, is the cause of diseases classified elsewhere
		B95.1	Specific	*Streptococcus*, group B, is the cause of diseases classified elsewhere
		B95.3	Specific	*Streptococcus pneumoniae* is the cause of diseases classified elsewhere
		B95.4	Specific	Other *Streptococcus* is the cause of diseases classified elsewhere
		B95.5	Specific	Unspecified *Streptococcus* as the cause of diseases classified elsewhere
		A40	Broad	Streptococcal sepsis
4	Enterococcus	B95.2	Specific	*Enterococcus* is the cause of diseases classified elsewhere
		A41.81	Specific	Sepsis due to *Enterococcus*
5	Hemophilus influenzae	B96.3	Specific	*Hemophilus influenzae* as the cause of diseases classified elsewhere
		A41.3	Specific	Sepsis due to *Hemophilus influenzae*
6	Klebsiella pneumoniae	B96.1	Specific	*Klebsiella pneumoniae* is the cause of diseases classified elsewhere
7	Escherichia coli	B96.2	Broad	*Escherichia coli* is the cause of diseases classified elsewhere
		A41.51	Specific	Sepsis due to *Escherichia coli*
8	Proteus	B96.4	Specific	*Proteus mirabilis, Proteus morganii* is the cause of diseases classified elsewhere
9	Pseudomonas	B96.5	Specific	*Pseudomonas aeruginosa*, *Pseudomonas mallei*, *Pseudomonas pseudomallei* as the cause of diseases classified elsewhere
		A41.52	Specific	Sepsis due to *Pseudomonas*

For bacteremia, two billing diagnostic codes are used simultaneously. For instance, if a patient has *Streptococcus* (group A) bacteremia, the billing code of R78.81 (for bacteremia) is combined with B95.0 (to specify the type of bacteremia).
